# Supplementary material for: Working from Home during the COVID-19 Pandemic and Its Effects on Diet, Sedentary Lifestyle, and Stress
Source: Nutrients. 2022 Sep 27;14(19):4006. doi: 10.3390/nu14194006 (PMC9572061; doi:10.3390/nu14194006)
Supplement: Supplementary file 1 [file nutrients-14-04006-s001.zip › nutrients-1877095-supplementary.pdf]

Supplementary Materials

Table S1. Changes in meal patterns after COVID-19 pandemic.

	**Before COVID-19**		**During COVID-19**		**p-value**
**Meals**	**n**	**%**	**n**	**%**	
Breakfast	264	80.5	257	78.4	0.473
Snacking Before Midday	242	73.8	183	55.8	**0.000***
Lunch	298	90.9	299	91.2	0.862
Snacking After Midday	45	13.7	77	23.5	**0.000***
Dinner	114	34.8	164	50.0	**0.000***
Snacking at Night	69	21.0	128	39.0	**0.000***

** $p<0.05$ Wilcoxon signed rank test*
